# Changes in the consumption of immediate-release fentanyl between 2018 and 2022 in the province of Santa Cruz de Tenerife after the implementation of regulatory measures by health authorities

**DOI:** 10.1007/s00210-026-05235-7

**Published:** 2026-04-09

**Authors:** María Rosario Pozuelo, Susana Abdala-Kuri, Alexis Oliva-Martín, Adama Peña-Vera, Chaxiraxi Morales-Marrero, Sandra Dévora-Gutiérrez

**Affiliations:** 1 Departamento de Medicina Física y Farmacología, Facultad de Farmacia, Universidad de La Laguna, Tenerife, Spain; 2 Departamento de Ingeniería Química y Tecnología Farmacéutica, Facultad de Farmacia, Universidad de La Laguna, Tenerife, Spain

**Keywords:** Immediate-release fentanyl, Transmucosal fentanyl, Wholesaler, Medication use, AEMPS safety communication, Prescription controlled system

## Abstract

In 2018, the Spanish Agency of Medicines and Medical Devices (AEMPS) issued a warning regarding the increasing use of immediate-release fentanyl (IRF) in Spain and recommended strict adherence to the approved indications. In April 2019, the regional government modified the electronic prescribing system to support this objective. In July 2021, the Ministry of Health implemented a restrictive prescribing and dispensing measure to further ensure compliance with the authorized indications. We analyzed trends in transmucosal IRF consumption in the province of Santa Cruz de Tenerife (SCTF) and across each of its constituent islands between 2018 and 2022. Additionally, we compared the evolution of IRF use in the province with national data for Spain. Data were obtained from pharmaceutical wholesalers supplying community pharmacies at the population level. IRF utilization was expressed as defined daily doses per 1000 inhabitants per day (DID). Linear regression models were applied to assess temporal trends. At baseline, IRF consumption in SCTF was approximately twice the national average. Marked heterogeneity in consumption patterns was observed across the different islands. Four distinct periods with differing trends were identified, three of which coincided with the implementation of new regulatory measures. Our findings indicate that annual IRF consumption in SCTF decreased by 54.76% between 2018 and 2022, representing a greater reduction than the national average. The 2018 warning had limited impact, whereas subsequent regional and national regulatory measures were associated with a substantial decline in IRF use.

## Introduction

Fentanyl is a major opioid analgesic (50–100 times stronger than morphine), classified at the third step of the World Health Organization (WHO) analgesic ladder, for the management of severe pain (Álamo et al. 2017; Anekar et al. 2025; Casely and Laycock 2022; González de Chávez 2025). In community pharmacies, it is available for both transdermal and transmucosal administration. Transdermal patches are long-acting and useful for the treatment of chronic oncologic and nononcologic pain, whereas transmucosal preparations are immediate-release formulations that provide rapid onset of analgesic action, making them suitable for the management of breakthrough cancer pain (BTcP) (De Rosa et al. 2023; Schug and Ting 2017).

BTcP differs from baseline cancer pain and requires distinct treatments (Greenfield et al. 2024; Løhre et al. 2016; Haugen et al. 2010; Jara et al. 2018; Mercadante and Portenoy 2016). Its prevalence among cancer patients ranges from 19 to 95% (Løhre et al. 2016; Jara et al. 2018; Batistaki et al. 2022; Cabezón-Gutiérrez et al. 2017; Cuomo 2023; Deandrea et al. 2014; Fallon et al. 2018; García-Benito et al. 2021; Jara-Sánchez and Beato-Zambrana 2020; López-Castro 2015; Mercadante 2015). Overall, it is estimated that 2 out of 3 patients with cancer pain will experience episodes of BTcP.

Transmucosal fentanyl fulfills the requirements for BTcP management and is used as rescue medication for such episodes (Álamo et al. 2017; Schug and Ting 2017; Cuomo 2023), in combination with long-acting opioid preparations (maintenance therapy) to control baseline pain (Jara et al. 2018; Jara-Sánchez and Beato-Zambrana 2020). In Spain, the authorized indication for transmucosal fentanyl is *the treatment of BTcP in adults receiving maintenance opioid therapy for baseline cancer pain, defined as patients taking at least 60 mg of oral morphine daily, 25 mg of transdermal fentanyl per hour, 30 mg of oxycodone daily, 8 mg of hydromorphone daily, or an equianalgesic dose of another opioid, for one week or longer* (AEMPS 2025). If a patient requires more than four rescue doses of IRF per day (more than four BTcP episodes), maintenance therapy should be reassessed (Batistaki et al. 2022; Fallon et al. 2018; AEMPS 2018a; Bañón-Morón et al. 2016).

Several studies have shown that many prescriptions do not comply with the authorized indication (*off-label* prescribing) (AEMPS 2018a; García-Sempere et al. 2022; González-Bermejo et al. 2021; Guastella et al. 2022; Jobski et al. 2023; Rollman et al. 2019; Weesie et al. 2023; Wirz et al. 2021). These include patients without a cancer diagnosis or with cancer who are not receiving baseline maintenance therapy. In Spain, in 2016, 40% of initial prescriptions for IRF in primary care were issued for noncancer pain (AEMPS 2018a).

Prolonged use of transmucosal fentanyl may result in opioid use disorder (tolerance, misuse, abuse, dependence, overdose, withdrawal syndrome, etc.) (AEMPS 2025). The risk is greater with higher doses and longer treatment duration (Casely and Laycock 2022; De Rosa et al. 2023; Blázquez-Puerta et al. 2020).

In Spain, nearly 60% of the cases of abuse and dependence reported to the Spanish Pharmacovigilance System (SEFV) as suspected adverse reactions involved patients using IRF for nonauthorized indications (AEMPS 2018a). In February 2018, the AEMPS issued a safety communication warning that overall fentanyl consumption within the Spanish National Health System (SNS) increased from 1.66 DID (defined daily doses per 1,000 inhabitants per day) in 2010 to 2.55 DID in 2016. For transmucosal fentanyl formulations, the increase during the same period more than doubled (from 0.16 to 0.41 DID) (AEMPS 2018a, 2025). At the same time, prescribers were reminded of *the importance of adhering to the authorized conditions of use to minimize the risk of abuse and/or dependence*.

Three years later, in July 2021, the Ministry of Health modified the prescription and dispensing requirements in the SNS by introducing a healthcare validation prior to dispensing to ensure compliance with the authorized indication (Montes-Gómez et al. 2021; Departament de Salut 2024).

The Canary Islands Health Service (SCS) had also warned in 2016 about the marked increase in consumption in the region (Bañón-Morón et al. 2016). In 2011, 11,000 packages were dispensed, compared with more than 46,000 in 2015, meaning that consumption tripled while the resident population increased by only 2.97% (National Statistical Institute 2024). Several regions adopted measures to curb this increase through diverse actions: periodic monitoring of patients using IRF at very high doses or as monotherapy, identification and review of patients with off-label prescriptions, educational activities, development and dissemination of guidelines and protocols for appropriate use, electronic prescribing alerts to physicians, limitations on use as chronic treatment, among others (AEMPS 2018a; Pharmacy Commission, Ministry of Health 2021).

Since 2016, SCS has implemented at least four such actions: three were informative (Bañón-Morón et al. 2016; Montes-Gómez et al. 2021; Caballero-Cabrera et al. 2016) and one, in April 2019, was more restrictive, consisting of changes to the prescribing module of the Canary Islands electronic prescribing system (REC) (Pharmacy Commission, Ministry of Health 2021; López-Navarro et al. 2020).

Although numerous national and international studies have examined opioid utilization in various countries over the past two decades (Jobski et al. 2023; Bosetti et al. 2019; Chenaf et al. 2019; Ju et al. 2022; Dart et al. 2021; García del Pozo et al. 2008; Gomes et al. 2022; Hamunen et al. 2009; Hastie et al. 2014; Häuser et al. 2021; Hurtado et al. 2022; Jarlbaek 2019; Hider-Mlynarz et al. 2018), few have focused on subnational levels (regional, provincial, insular, and municipal) (González de Chávez 2025; Oliva et al. 2023, 2022; Hurtado et al. 2020; Torres-Bueno et al. 2024; Xie et al. 2022). Even fewer have analyzed IRF consumption specifically (González-Bermejo et al. 2021; Arrieta-Loitegui et al. 2020; Fleischman et al. 2019), and very few have investigated the impact of regulatory interventions by health authorities on IRF use (García-Sempere et al. 2022; Rubio-Esparza et al. 2025).

The use of transmucosal IRF has increased considerably in Spain in recent years, prompting the implementation of several measures aimed at minimizing risks for patients. However, few studies have evaluated the impact of these interventions on prescribing patterns.

The objective of this study is to analyze trends in consumption of transmucosal IRF, affected by regulatory actions of health authorities, dispensed on each island and in the province of Santa Cruz de Tenerife as a whole, between 2018 and 2022. Secondly, we aim to compare consumption patterns in the province with national patterns. Finally, we intend to compare the evolution of IRF use with cancer mortality rate in the province.

## Materials and methods

### Data

This work was carried out using the methodology of drug utilization studies (DUS). For this purpose, we used the ATC/DDD system, which applies the anatomical therapeutic chemical (ATC) classification and the defined daily dose (DDD) as the unit of measurement to quantify medication consumption (AEMPS 2018b; WHO Collaborating Centre for Drug Statistics Methodology 2024). The DDD of transmucosal IRF is 0.6 mg, a value obtained from the *ATC/DDD Index 2025* published on the website of the WHO Collaborating Centre for Drug Statistics Methodology, https://atcddd.fhi.no/ (WHO Collaborating Centre for Drug Statistics Methodology 2023, 2024).

In this study, medicine consumption was expressed as defined daily doses per 1000 inhabitants per day (DID), which represents the number of DDDs consumed per 1000 inhabitants per day. This indicator provides an approximate estimate of the proportion of the population treated daily with a standard dose of a given medication and allows for comparisons of drug utilization across different populations and within the same population over time (Laporte and Tognoni 1993; AEMPS 2018b).

Medicines marketed in Spain containing transmucosal IRF were obtained from the BOT PLUS database (Consejo General de Colegios Farmacéuticos 2024).

The study of transmucosal IRF use in the province was carried out based on the number of medicine packages supplied to community pharmacies by authorized distribution entities located in the province of Santa Cruz de Tenerife (SCTF), namely, COFARTE (Cooperativa Farmacéutica de Tenerife) and COFARES (Cooperativa Farmacéutica Española). Both cooperatives provided data on the packages supplied during the study period, including the supply date and the pharmacy postal code (in compliance with Spanish data protection legislation) (Data Protection Law in Spain 2018). Identification by postal code enabled comparison of consumption among the different islands within the province of SCTF. The data included medicines dispensed in the public sector (SNS, mutualities MUFACE, ISFAS, and MUGEJU) and the private sector; hospital consumption was not included.

Permanent resident population was obtained from the National Institute of Statistics (INE) (Consejo 2024).

We analyzed the evolution of consumption of these medicines from January 1, 2018 (baseline) to December 31, 2022 (final).

To determine national consumption and compare it with provincial data, we used the most recent report on *Utilization of opioid analgesic medicines in Spain*, published on October 28, 2025, by the Observatory on Medicine Use of the AEMPS. This report shows national IRF consumption since 2010, broken down by route of administration, nasal and sublingual/buccal (AEMPS 2026).

Cancer mortality rate in the province, to be compared with IRF consumption trends, was obtained from the Spanish Association Against Cancer (AECC 2026). These data come from GLOBOCAN, the database of the Global Cancer Observatory (GCO), under the auspices of the WHO (Global Cancer Observatory 2025; Bray et al. 2024).

### Statistical analysis

We used the linear regression model to fit time trends. Before fitting all regression models, data were visualized graphically to identify general trends and potentially influential outliers. The specified interventions were as follows:

- AEMPS communication in February 2018
- Change in REC in April 2019
- Prescription controlled system in July 2021

The general equation of the regression model corresponds to

$$Y\left(ij\right) = {\beta }_{0} + {\beta }_{1} \cdot t + {e}_{\left(ij\right)}$$

where *Y*(*ij*) is the dependent variable indicating the monthly DID per 1000 patients at time *t*; *t* is a discrete variable representing time in months, assigned as a sequential number starting from 1 (corresponding to the first month) and increasing incrementally up to the total number of quarters in the study period; ${\beta }_{0}$ estimates the baseline outcome (DID of drug use per months at time 0); ${\beta }_{1}$, estimates the baseline slope, that is, the secular trend in DID before the intervention. The slope is the change in the outcome value per unit of change in the time value. For this study, the slope represents the change in DID per months; and $\varepsilon$ is the error term assuming it is independent following a normal distribution with zero mean and constant variance (*N* ~ (0, ${\sigma }^{2}$).

**Fig. 1:**
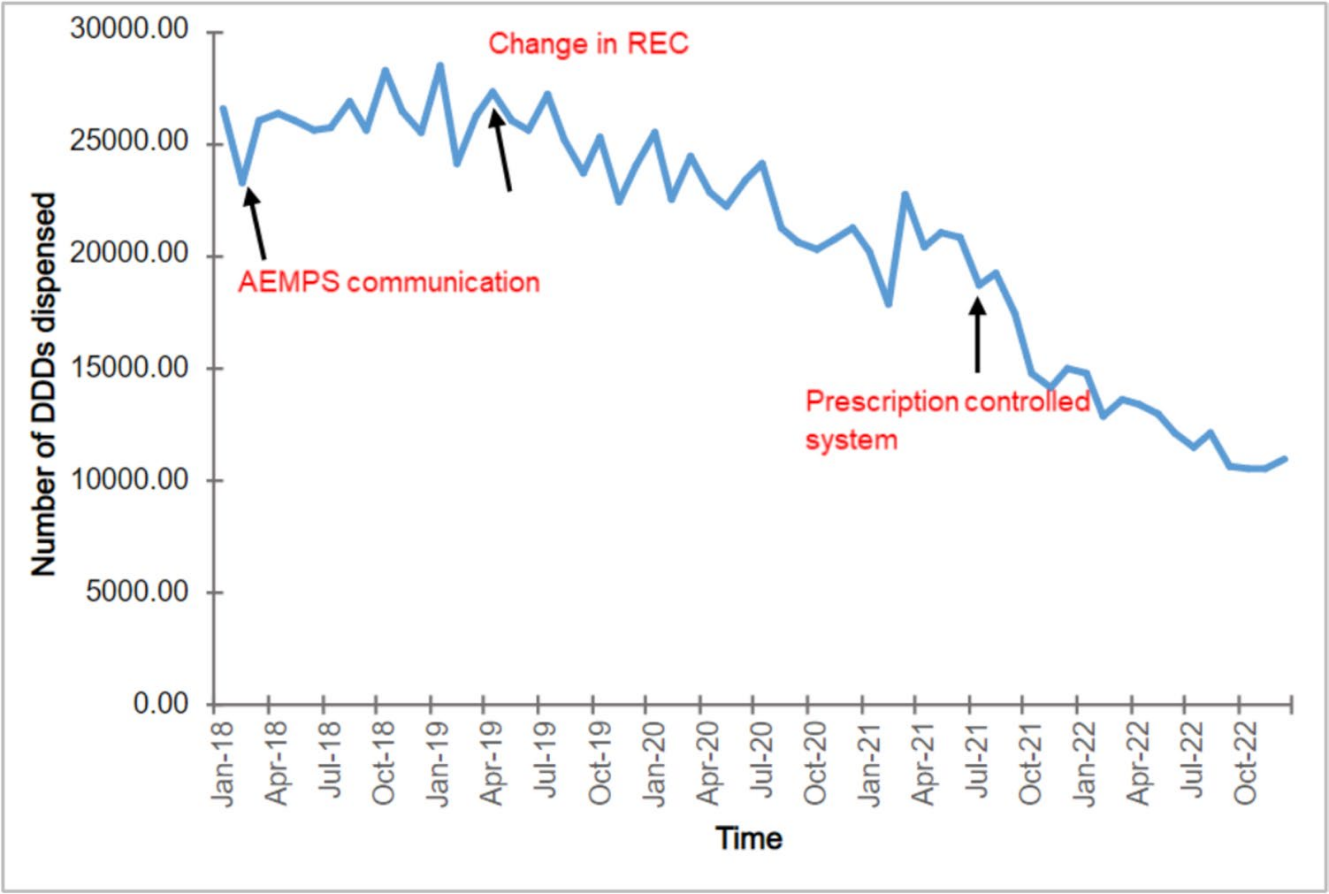
Evolution of monthly IRF consumption in the province of SCTF during the period 2018–2022, expressed as the number of DDDs supplied to pharmacies. DDDs: defined daily doses. AEMPS: Spanish Agency of Medicines and Medical Devices. REC: Canary Islands electronic prescribing system

**Fig. 2:**
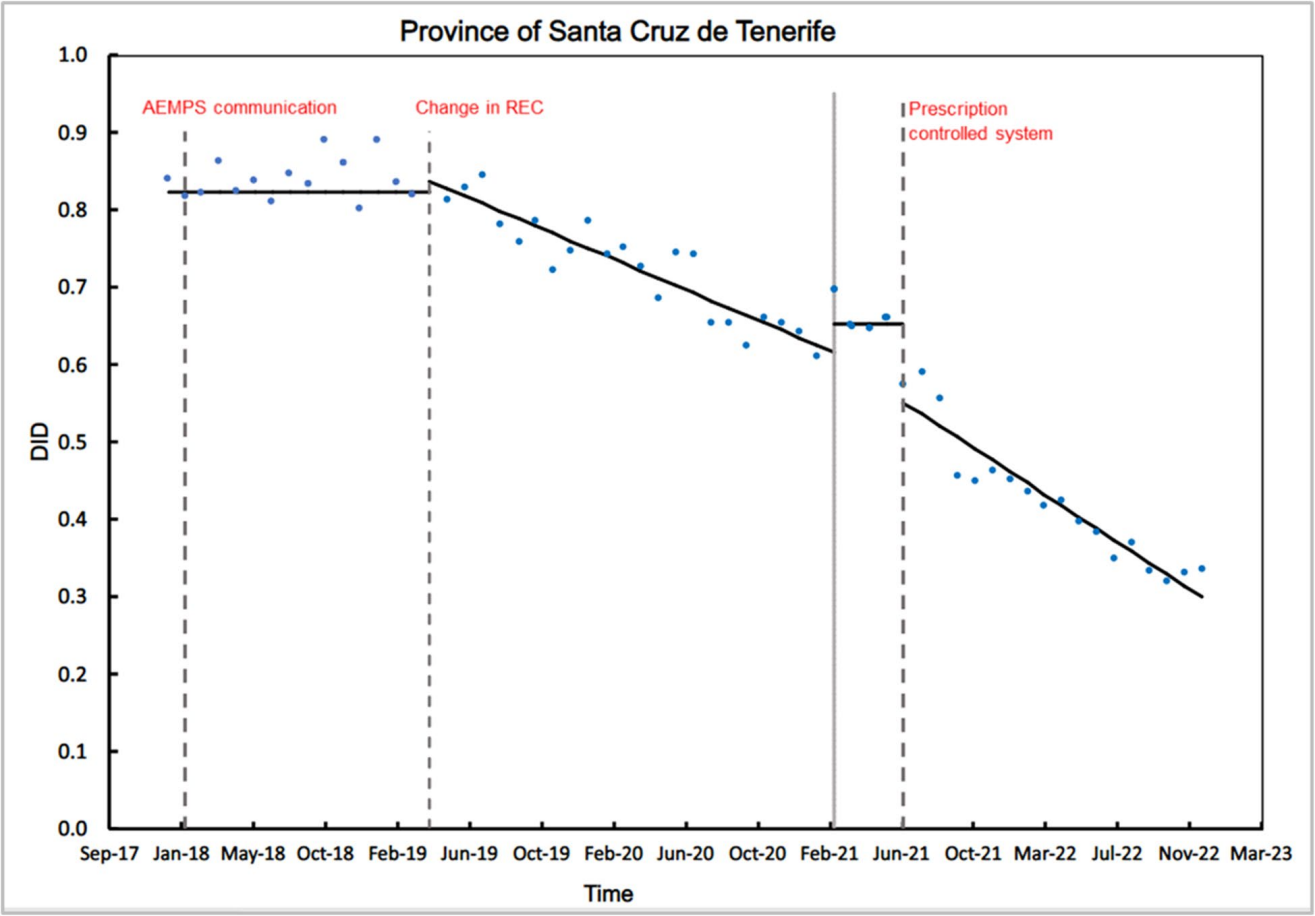
Linear regression model of monthly immediate-release fentanyl consumption expressed as DID (defined daily doses per 1000 inhabitants per day) between 2018 and 2022. The solid black lines represent the DID values estimated by the linear regression model for each period. The vertical dashed lines indicate the inflection points, three of which temporally coincided with regulatory interventions

**Fig. 3:**
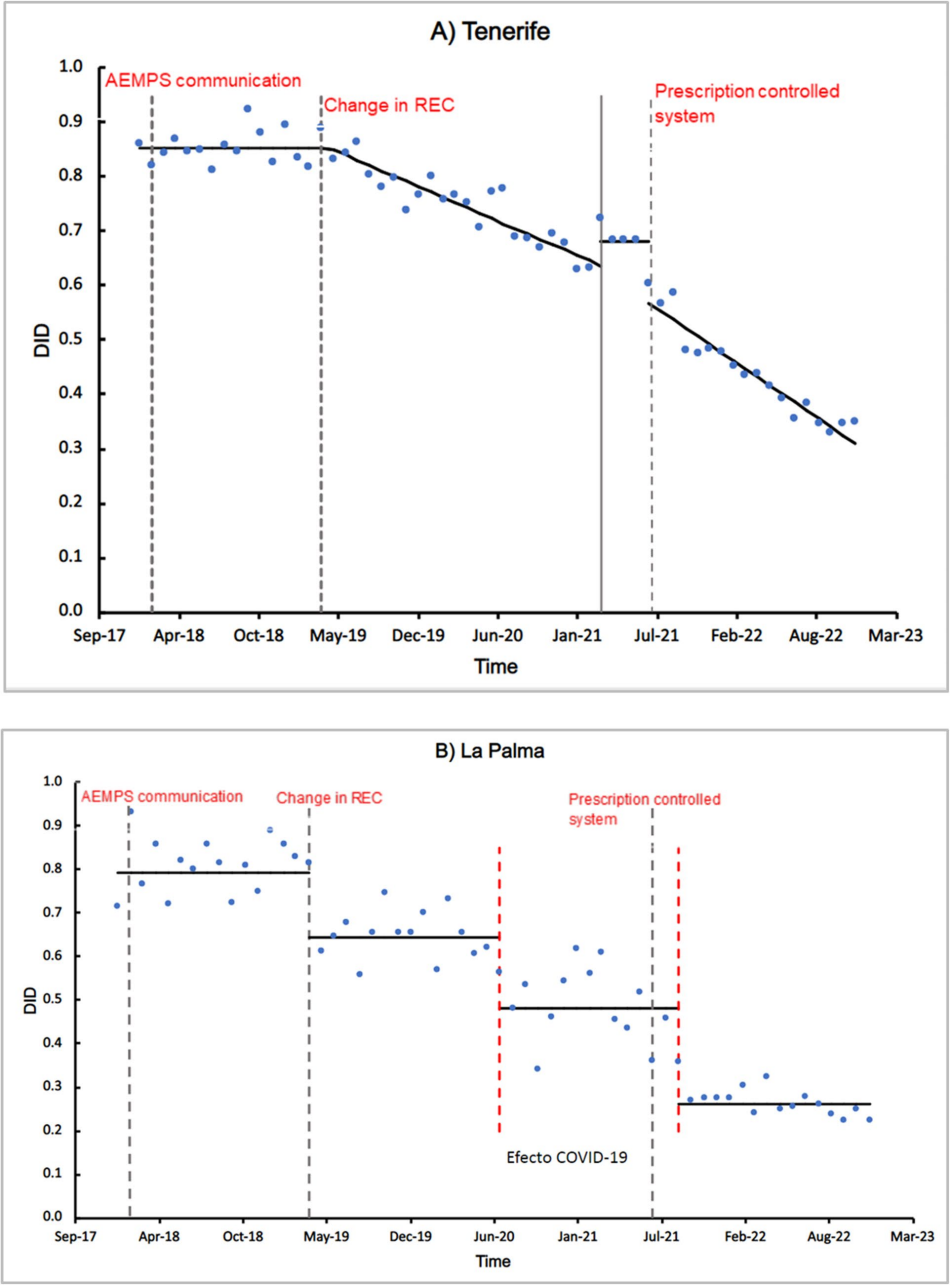

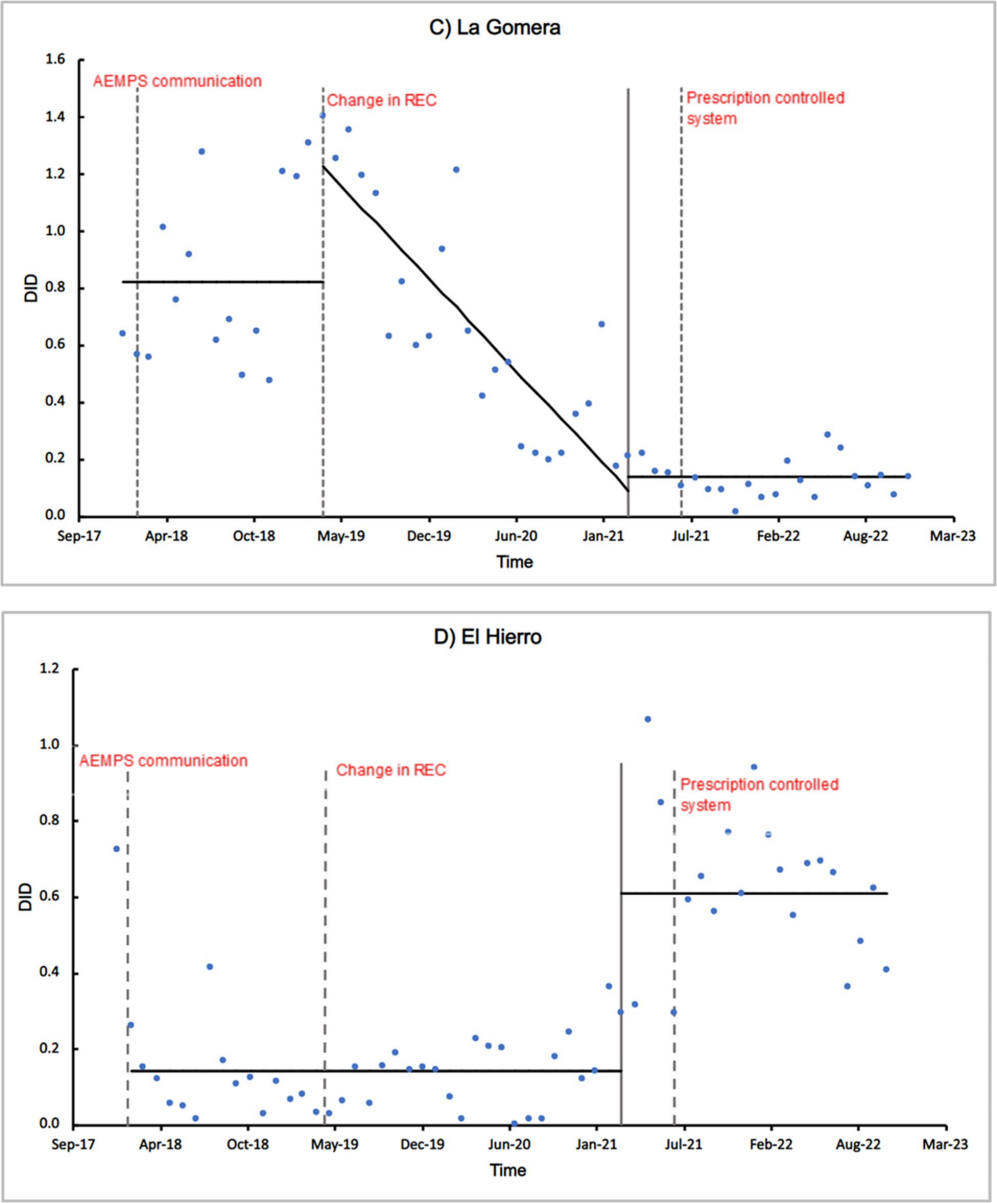
Analysis of consumption trends observed for each island within the province of SCTF

Results of the final model in each period are presented in Table 2, with 95% confidence intervals (95% CI) between brackets, and in Figs. 1, 2 and 3. R software version (4.1.2) Copyright (C) 2021 was used for statistical analysis.

## Results

The province of SCTF is one of the two provinces of the Canary Islands (Spain) and is composed of four islands: Tenerife, La Palma, La Gomera, and El Hierro. Tenerife accounts for 89% of the total population of the province (approximately 934,000 inhabitants in 2022), compared with 8% in La Palma (Ca. 84,000), 2% in La Gomera (22,000), and 1% in El Hierro (11,000) (National Statistical Institute 2024).

The list of medicines authorized in Spain containing IRF included 125 presentations of different commercial brands, doses, package sizes, and marketing dates (Table 1).

**Table 1:** List of medicines containing immediate-release fentanyl (IRF), authorized in Spain

Name	Fentanyl content of different formats	Pharmaceutical form	Route of administration	Marketing date
ABFENTIQ® EFG	200, 400, 600 and 800 mcg	Oral transmucosal lozenge	Transmucosal buccal	2018
ABSTRAL®	100, 200, 300, 400, 600, and 800 mcg	Sublingual tablets	Transmucosal sublingual	2009
ACTIQ®	200, 400, 600, 800, 1200, and 1600 mcg	Oral transmucosal lozenge	Transmucosal buccal	2011
AVARIC®	67, 133, 267, 400, 533, and 800 mcg	Sublingual tablets	Transmucosal sublingual	2014
BREAKYL®	200, 400, 600, 800, and 1200 mcg	Buccal soluble film	Transmucosal buccal	2014
EFFENTORA®	100, 200, 400, 600, and 800 mcg	Buccal tablets	Transmucosal buccal	2009
FENTICERTA® EFG	100, 200, 300, 400, 600, and 800 mcg	Sublingual tablets	Transmucosal sublingual	2020
KAPTIC® EFG	100, 200, 300, and 400 mcg	Sublingual tablets	Transmucosal sublingual	2020
INSTANYL®	50, 100, and 200 mcg	Nasal spray	Intranasal	2013
PECFENT®	100 and 400 mcg	Nasal spray	Intranasal	2011

The number of DDDs dispensed in SCTF pharmacies at baseline was 26,568 (Fig. 1). In the following six months, fluctuations were observed around this figure. In 2019, there were months in which sales exceeded those of the first month (28,466 DDDs in January, 27,302 in April, and 27,174 packages in July 2019), although a downward trend began to be perceived, becoming more pronounced from the second half of that year onward. By the end of the study period, the number of DDDs dispensed had decreased to 10,992.

The monthly consumption profile of IRF differed across the four islands (Table 2).

**Table 2:** Impact of the regulatory measures on immediate release fentanyl monthly consumption expressed in DID (DDDs per 1,000 inhabitants and day)

	Period 1	Period 2	Period 3	Period 4
Province of SCTF				
Level of consumption (intercep)	0.823 [0.789; 0.857] (*n* = 16)	0.837 [0.812; 0.862] (*n* = 22)	0.653 [0.515; 0.770] (*n* = 4)	0.556 [0.536; 0.594] (*n* = 18)
Variation rate (slope)	0.00248 [− 0.00103; 0.0060]*	− 0.00965 [− 0.0116; − 0.00771]	0.00531 [− 0.0538; 0.0644]*	− 0.0148 [− 0.0175; − 0.0121]
Use (%)	-	− 25.3%	+ 6.18%	− 14.8%
Tenerife				
Level of consumption (intercep)	0.847 [0.817; 0.876] (*n* = 16)	0.868 [0.843; 0.893] (*n* = 22)	0.680 [0.129;1.024] (*n* = 4)	0.582 [0.556; 0.609] (*n* = 18)
Variation rate (slope)	0.000492 [− 0.00212; 0.00310]*	− 0.00967 [− 0.0115; − 0.00784]	0.0293 [− 0.193; 0.134]*	− 0.0151 [− 0.0175; − 0.0127]
Use (%)		− 25.6%	+ 1.08%	− 14.4%
La Palma				
Level of consumption (intercep)	0.792 [0.720; 0.864] (*n* = 16)	0.691 [0.619; 0.714] *(n* = 15)	0.480 [0.428; 0.534] (*n* = 13)	0.290 [0.262; 0.317] (*n* = 16)
Variation rate (slope)	0.00189 [− 0.00556; 0.00935]*	− 0.00807 [− 0.0136; − 0.00252]	− 0.00464 [− 0.0169; 0.00766]*	− 0.00348 [− 0.00608; 0.00456]*
Use (%)	-	− 12.7%	− 30.5%	− 39.6%
La Gomera				
Level of consumption (intercept)	0.603 [0.268; 0.933] (*n* = 16)	1.227 [1.036; 1.419] (*n* = 23)	0.142 [0.0801; 0.202] (*n* = 21)	
Variation rate (slope)	0.0277 [− 0.00889; 0.0643]*	− 0.0494 [− 0.0643; − 0.0344]	− 0.000761 [− 0.00538; 0.00386]*	
Use (%)	-	+ 92.5%	+ 54.3%	
El Hierro				
Level of consumption (intercept)	0.142 [0.0821; 0.265] (*n* = 39)		0.606 [0.590; 0.784] (*n* = 21)	
Variation rate (slope)	− 0.00162 [− 0.00570; 0.00246]*		− 0.0131 [− 0.0283; 0.00217]*	
Use (%)	-		+ 330%	

At the provincial level, four distinct periods were clearly identified. Three of them (the first, second, and fourth) were associated with the implementation of health authority measures, whereas the third period appeared unrelated to these interventions.

In the first segment, consumption remained stable at 0.823 DID, with a nonsignificant slope, as the 95% confidence intervals included zero. In the second segment, a downward trend was observed, with a monthly rate of change of − 0.00965 DID and an estimated consumption level of 0.615 DID at the end of the period.

The third segment, which lasted only four months and was the shortest analyzed period, showed stable consumption at 0.653 DID, slightly higher than the level estimated at the end of the second segment.

Finally, in the fourth segment, consumption declined again at a monthly rate of − 0.0148 DID, reaching an estimated level of 0.299 DID at the end of the study period.

For the island of Tenerife, the same pattern and trends observed at the provincial level were identified (Table 2 and Figs. 2 and 3A). Furthermore, tests for equality of slopes and intercepts applied to the second and fourth segments confirmed the absence of statistically significant differences (*p* > 0.05).

On the island of La Palma, the first segment showed a pattern similar to that observed in Tenerife, with an estimated consumption rate of 0.792 DID, slightly lower than that of Tenerife (0.847 DID). In the fourth segment, consumption remained stable at 0.290 DID, with a nonsignificant slope (Table 2).

However, the second and third segments displayed trends that differed from those observed in other geographical settings. An initial analysis combining both segments into a single period suggested that the linear model was not appropriate, as reflected by a low coefficient of determination (*R*^2^ = 0.28), indicating poor goodness-of-fit. Visual inspection of the data (Fig. 3B) suggests the presence of two overlapping clusters between the second and third segments, supporting subdivision into two subperiods: the first from April 2019 to June 2020, and the second from July 2020 to August 2021 (one month after the July 2021 restrictive measure, as consumption values during that month fell within the variability range of the first sub-period). Both sub-periods showed stable consumption, although at a higher level in the first (0.653 DID) compared with the second (0.480 DID).

For La Gomera, the first segment showed stable consumption similar to that observed on the other islands, with an estimated mean rate of 0.603 DID, albeit with considerable variability, as reflected by the wide 95% confidence interval [0.268; 0.933] (Table 2).

The second segment exhibited a marked decline in consumption, starting from a very high estimated level of 1.227 DID (approximately double the mean observed in the first segment), with a monthly rate of change of − 0.0494 DID. This slope was approximately five times steeper than that observed for Tenerife and La Palma, reaching an estimated level of 0.092 DID at the end of the period.

The third segment showed stable consumption at 0.183 DID, approximately double the level observed at the end of the second segment. This stable pattern persisted during the fourth segment (0.121 DID), although at a lower level than at the beginning of the third segment.

When segments three and four were combined—given their similar variability, a situation analogous to that observed in El Hierro—the estimated mean consumption was 0.142 DID, with a nonsignificant slope (− 0.000761 DID per month; 95% CI: − 0.00538 to 0.00386) (Table 2).

In El Hierro, consumption remained stable during the first segment, with a nonsignificant slope (Table 2). Unlike Tenerife and La Palma, this stable pattern extended until February 2021. A combined analysis of the first and second segments yielded a mean consumption of 0.142 DID, with a nonsignificant slope (*p* > 0.05).

However, mean consumption differed markedly between the periods before and after March 2021, increasing from 0.142 DID to an estimated mean of 0.611 DID—more than a fourfold rise—despite the restrictive intervention implemented in July 2021.

**Fig. 4:**
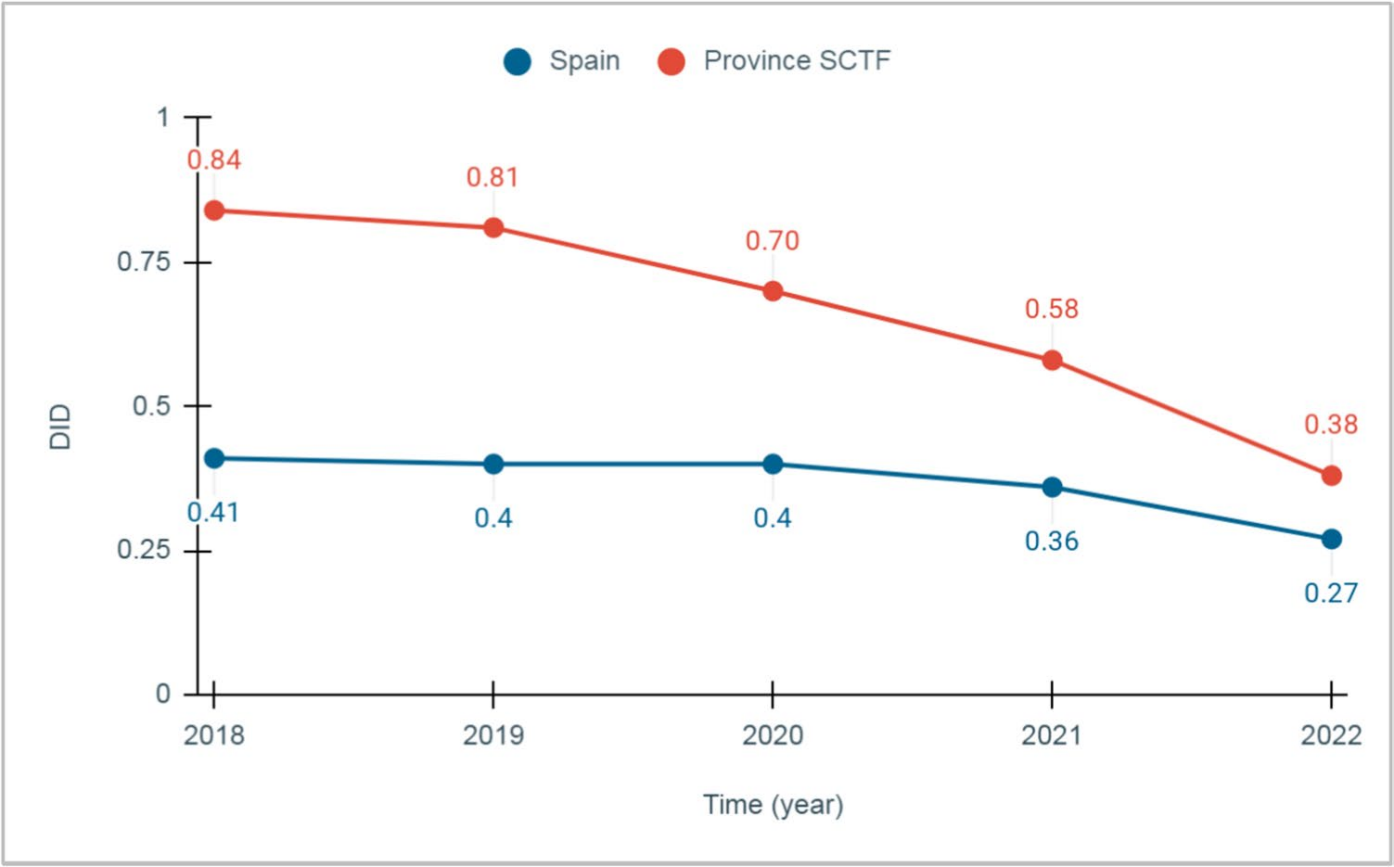
Comparison of the evolution of annual IRF consumption in Spain and in the province of SCTF between 2018 and 2022

When annual consumption in the province was compared with the national level, provincial consumption was consistently higher throughout the study period—more than double at baseline (0.84 DID vs. 0.41 DID) and 41% higher at the end of the study (0.38 DID vs. 0.27 DID) (Fig. 4).

**Fig. 5:**
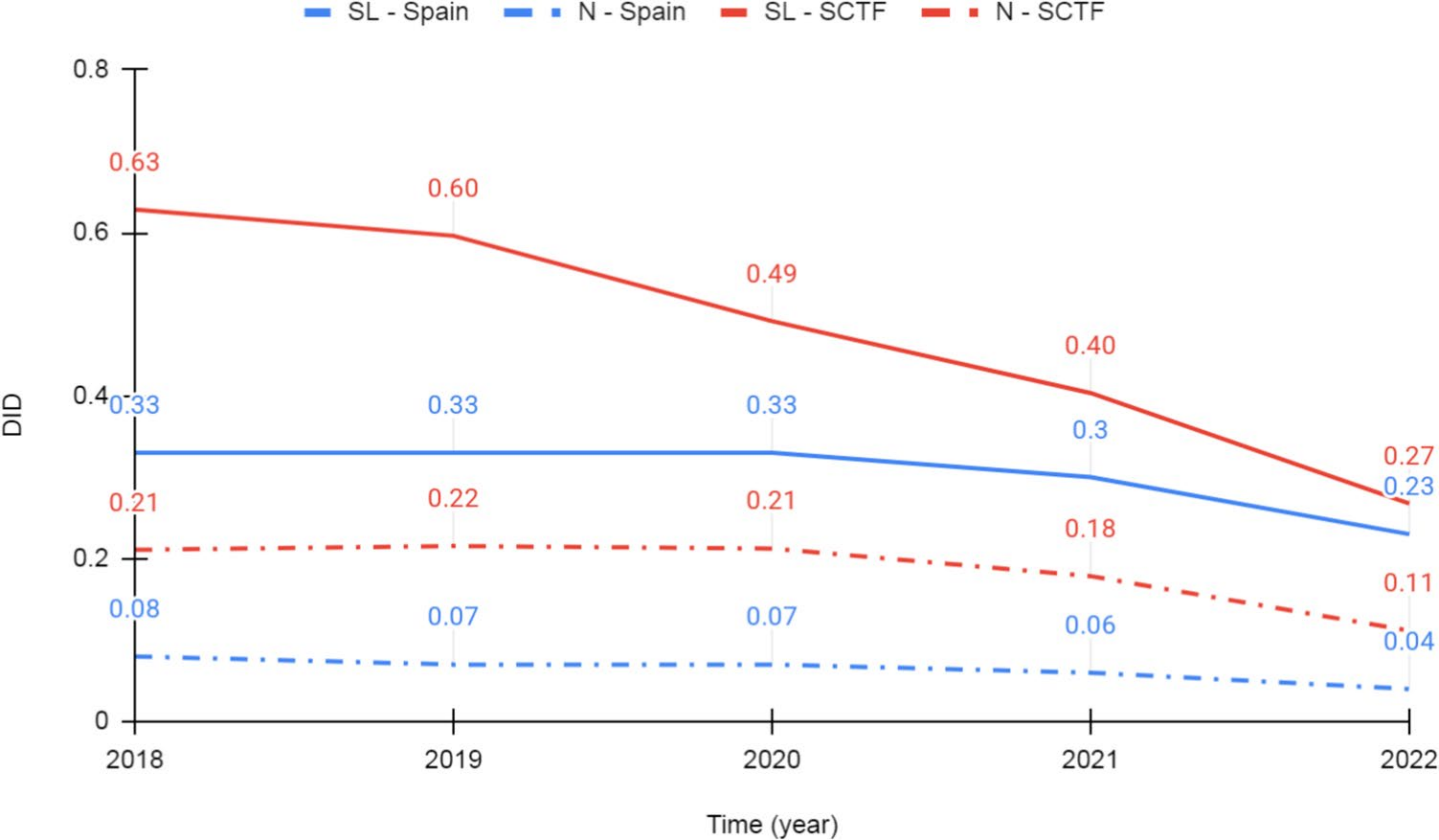
Evolution of annual IRF consumption by route of administration, national versus the province of SCTF. DID: Defined daily doses per 1,000 inhabitants per day. N: nasal route. SL: sublingual, buccal, oral route

In both Spain and the province, the use of orally administered formulations was substantially higher than that of nasal formulations (in the province, 0.63 and 0.21 DID, respectively; nationally, 0.33 and 0.08 DID, respectively) (Fig. 5). All presentations showed a decline (in the province, 57.14% for oral and 47.62% for nasal formulations; nationally, 30.3% and 50%, respectively).

**Fig. 6:**
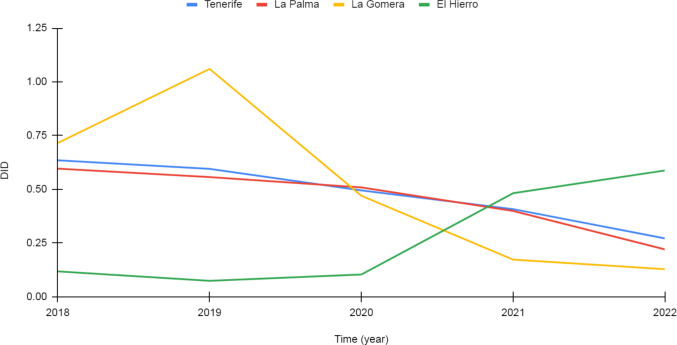
Evolution of annual IRF consumption via sublingual, oral, and buccal administration, by island (in DID). DID: Defined daily doses per 1000 inhabitants per day

Regarding the evolution of oral formulations (Fig. 6), the islands of Tenerife and La Palma showed a fairly similar downward trend. In contrast, La Gomera presented a consumption peak one year after the 2018 alert (from 0.72 DID in 2018 to 1.06 in 2019), meanwhile El Hierro increased its consumption (from 0.12 DID in 2018 to 0.59 in 2022) despite the regulatory measures.

**Fig. 7:**
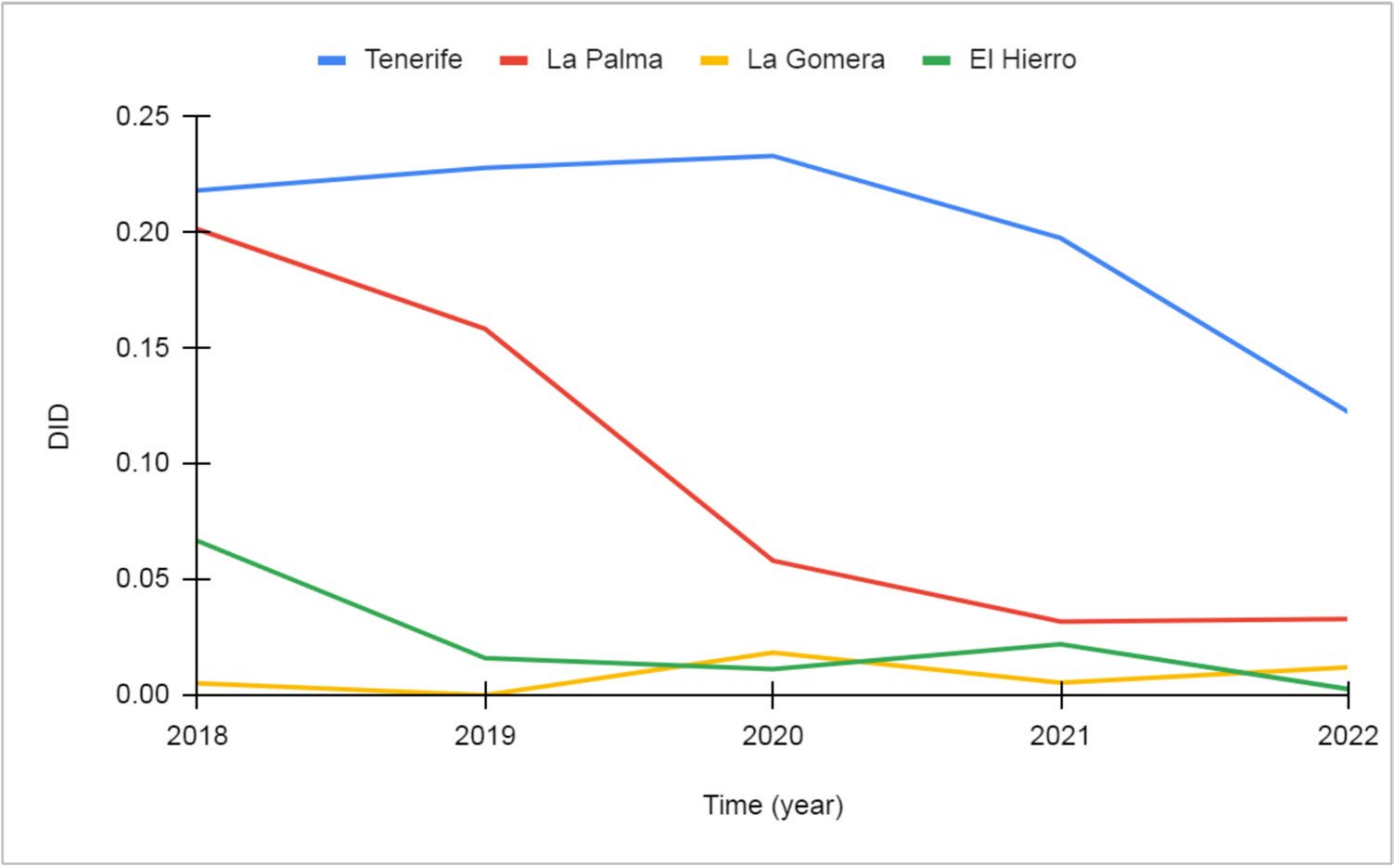
Evolution of IRF consumption via nasal administration by island (in DID). DID: defined daily doses per 1000 inhabitants per day

With regard to nasal formulations, the four islands showed different patterns (Fig. 7). However, the net result was that all, except La Gomera, reduced their consumption by the end of the study period (Tenerife decreased by almost 44%, La Palma by around 84%, La Gomera increased by 134%, and El Hierro reduced by 96%).

**Fig. 8:**
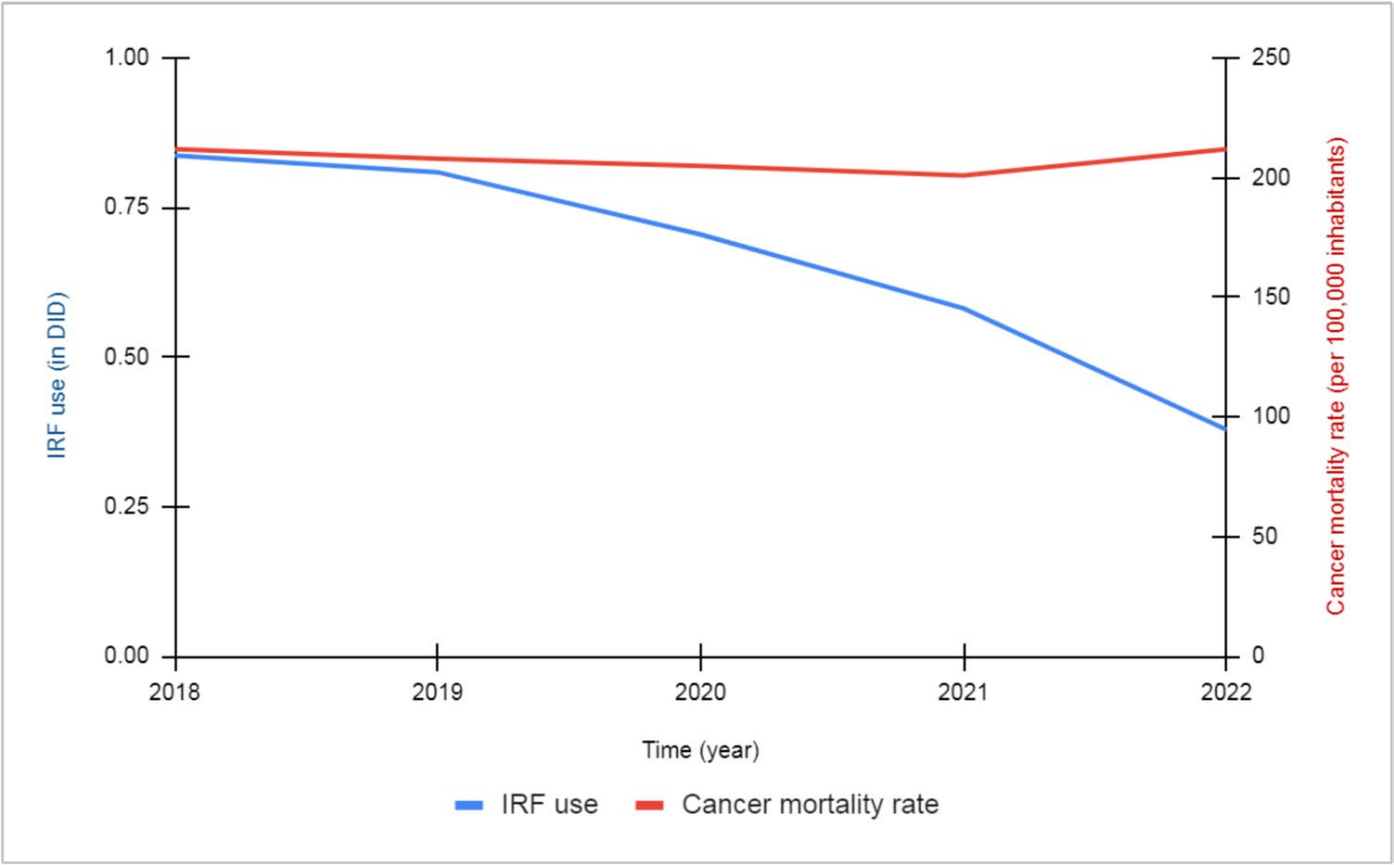
Evolution of IRF consumption and cancer mortality rate in the province of SCTF between 2018 and 2022. DID: Defined daily doses per 1000 inhabitants per day. IRF: Immediate-release fentanyl. SCTF: Santa Cruz de Tenerife

Finally, it was observed that the cancer mortality rate in the province of SCTF remained almost flat, while annual IRF consumption decreased by 54.76% by the end of the study (Fig. 8).

## Discussion

When a drug safety issue is detected, health authorities and pharmaceutical companies are required to take the necessary measures to reduce the risks associated with its use. These measures may vary, being more or less restrictive depending on the severity of the risk. They range from simply informing about the newly identified safety issue to the immediate withdrawal of the drug from the market, with a spectrum of intermediate actions such as patient monitoring, restriction of indications, limitation to specific population groups, restriction of the prescribing setting, gradual withdrawal, and others (Abajo-Iglesias et al. 2003; Rosich-Martí 2020). In general, these measures are effective, and after their dissemination, a reduction in prescribing or at least a modification in consumption trends is observed. However, after this initial caution, the effect tends to fade over time, returning to the initial situation, and in some cases, successive alerts are required to achieve a reduction in consumption consistent with the restrictions proposed by the authorities (Hoffman et al. 2014; Iglesias-Carbajo 2017). In our study, we observed a limited effect in the two semesters following the initial alert, and only when new, more rigorous measures were implemented (April 2019 and July 2021) did the effect become immediate and substantial (Figs. 1 and 2 and Table 2).

Numerous factors are likely to influence the response to a safety alert: the severity of the adverse effect, whether the new instructions or recommendations entail a greater workload for prescribers, the degree of dissemination of the alert, whether the scientific basis of the warning is supported by strong evidence, and whether the alert includes information on safer alternatives (Hoffman et al. 2014). The impact is smaller if the initial prescription must be made by a specialist (Reber et al. 2013); prescriber inertia to avoid intervention (Rosich-Martí 2020; Anderson et al. 2020), as well as the level of training in pain management, may also play a role. Neither the emergence of new scientific evidence, however robust, nor the dissemination of pharmaceutical alerts necessarily translates into an automatic change in clinical practice, as multiple factors (physician, patient, specialists, experts, industry, media, and healthcare organization) may influence prescribers’ implementation of such recommendations (Rosich-Martí 2020).

The 2018 safety alert (AEMPS 2018a) initially produced in the province of SCTF a response contrary to what was expected, since the number of DDDs dispensed in the second half of 2018 increased by 2.84% compared to the previous semester. This measure merely provided recommendations to physicians to adjust prescribing to the authorized indication; it did not constitute a mandatory requirement to direct prescribing toward other therapeutic alternatives, which is reflected in the poor adherence observed, as we have confirmed (Fig. 2).

Although these IRF formulations were first marketed in Spain in 2009 (Consejo General de Colegios Farmacéuticos 2024), some products were introduced more recently (Instanyl® nasal spray in 2013; Breakyl® buccal soluble film and Avaric® sublingual tablets in 2014; and Abfentiq® oral transmucosal lozenge in 2018) (Table 1). Consequently, part of the observed increase may reflect a typical post-marketing expansion phase, during which healthcare professionals progressively gain familiarity with the product, its indications, and clinical use. This dynamic could have counteracted the intended effect of the AEMPS warning (Rubio-Esparza et al. 2025).

Furthermore, in Spain, prescriptions for narcotic medicines may cover up to a three-month treatment period (Ministry of the Presidency 2012). Therefore, a latency period of similar duration could be expected before any regulatory measure produces a measurable impact. However, in this case, the anticipated decline in consumption was not observed in the subsequent months.

In April 2019, the Canary Islands Health Administration implemented a more restrictive measure, requiring that new IRF prescriptions be explicitly linked to the authorized indications specified in the summary of product characteristics (López-Navarro et al. 2020). Our study showed that the number of DDDs dispensed in the province in the second half of 2019 decreased by 6.31% compared with the previous semester, a decline that continued over the next three semesters. The SCS acknowledged that this action had a visible impact on consumption, as it resulted in a 14.25% decrease in the number of patients treated with IRF in October 2019 compared with October 2018, as well as an 8.22% reduction in the number of packages dispensed at the regional level (Pharmacy Commission, Ministry of Health 2021). We observed a reduction of 8.94% in the number of packages dispensed during the same months in the province of SCTF.

The last three six-month periods of the study showed a marked decrease compared with the immediately preceding semester (− 19.31%, − 19.75%, and − 16.93%, respectively). The most likely cause of this decline was the entry into force, on July 1, 2021, of the decision by the Ministry of Health to establish a special restriction within the SNS for transmucosal IRF formulations. This restriction (Ministry of Health 2007) consisted of the implementation of a *prescription-controlled system* (or health validation) prior to dispensing, for new treatments with these medicines. Treatments initiated before this date, which were maintained at the clinician’s discretion, were not affected by the health validation requirement. However, in the specific context of the Canary Islands, the REC system only allowed dose reductions, not increases (Montes-Gómez et al. 2021; Departament de Salut 2024). It appears that the impact of the 2018 AEMPS alert in Spain as a whole was limited, which led to the approval in 2021 of a stricter measure, namely the imposition of a prescription-controlled system, which produced a marked reduction in consumption from that moment onward at the national level. In this regard, the work of Rubio-Esparza et al. (2025) confirms this idea, showing that the impact of the 2021 measure was considerably more effective in reducing consumption than the 2018 measure.

It is noteworthy that a few months prior to the implementation of the regional and national regulatory interventions, a temporary increase in monthly consumption was observed, the cause of which remains unclear. At the regional level, modifications to the electronic prescribing system (REC) are typically communicated in advance to prescribing physicians to allow for implementation. Similarly, at the national level, the Ministry of Health generally informs regional authorities approximately three months before introducing new regulatory measures, and these authorities subsequently notify the affected healthcare professionals. It remains uncertain whether the observed increase was a random fluctuation or the result of a specific event that influenced prescribing behavior.

The monthly comparative analysis between the island of Tenerife and the province as a whole (i.e., the four islands combined) indicates no statistically significant differences between these geographical settings, as confirmed by the tests for equality of slopes and intercepts (*p* > 0.05) performed for segments 2 and 4. This finding may be explained by the fact that Tenerife accounts for 88.8% of the province’s total population, compared with 1.08% for El Hierro and 2.08% for La Gomera.

At the provincial level, monthly consumption decreased by 63.7% between January 2018 and December 2022, although the magnitude of the decline varied across segments. The largest reduction occurred during the second segment (25.3%), whereas the decrease during the fourth segment was more moderate (14.8%). In contrast, a slight increase of 6.18% was observed during the third segment, approximately six percentage points higher than that observed in Tenerife.

The difference between segments 2 and 4 can be attributed to the slope of the regression lines, which was 1.5 times steeper in the second segment than in the fourth (− 0.0151 vs. − 0.00965 DID per month). This suggests that the effect of the regional REC-related measures may have been greater than that of the subsequent national prescription-controlled system.

On the island of El Hierro, two clearly differentiated periods of stable consumption were identified (Fig. 3D), although at markedly different levels. Between January 2018 and December 2022, consumption increased by 330%. In the first segment, consumption levels were low compared with the other islands (0.142 DID vs. a mean of 0.800 DID), whereas in the final segment consumption rose to 0.611 DID, substantially exceeding the levels observed elsewhere (Table 2). A plausible explanation for this variability may lie in the small population size (approximately 11,000 inhabitants), where the addition or withdrawal of a small number of patients can markedly influence the calculated DID values.

Throughout the study period, no changes occurred in the island’s public healthcare infrastructure (one hospital in the island capital and six primary care centers distributed across the territory), nor were new healthcare services introduced that might account for the increase in prescribing and consumption.

The results for La Gomera showed a pattern broadly similar to that observed in Tenerife, although consumption during the third and fourth segments may be considered stable, with an estimated mean of 0.142 DID. However, notable differences were observed. During the first segment, mean consumption was 0.603 DID with a coefficient of variation of 25%, followed by a 28.8% decline. By the end of this period, estimated consumption had doubled, reaching 1.227 DID. As shown in Fig. 3C, variability during the first segment was high (coefficient of variation: 25%), with a marked upward shift in consumption during the final third of the segment, the cause of which remains unknown.

Following the regional intervention, a sharp decline in consumption was observed, with a slope of − 0.0494 DID per month—approximately five times steeper than that observed in Tenerife and six times that observed in La Palma (Table 2). The reasons for this decline remain uncertain (e.g., potential impact of the COVID-19 pandemic, a reduction in the number of treated patients, or stricter prescribing control after the implementation of the measure). The latter explanation appears more plausible, as consumption during the final segment remained stable and slightly above that observed at the end of the third segment, suggesting tighter control of prescribing and a stable number of treated patients.

Oliva (2022) examined patterns of opioid use on La Gomera between 2016 and 2019 and concluded that factors such as population aging, socioeconomic status, access to healthcare services, and rural–urban differences did not account for opioid prescribing rates. The author further noted that opioid therapy is initiated by specialist physicians at the island’s only hospital, which may facilitate appropriate indication and dosing. This could explain the overall opioid prescribing rates observed in La Gomera; however, it does not account for the high IRF consumption observed during the final third of the first segment.

La Palma exhibited a pattern clearly distinct from that observed on the other islands and at the provincial level. A stepwise decline in consumption was observed throughout the study period, with relatively stable levels within segments. Consumption decreased from 0.792 DID in January 2018 to 0.290 DID at the end of the study period, representing a 63.4% reduction—similar to that observed at the provincial level.

The first and fourth segments coincided with those identified in the other geographical areas, whereas the second and third segments followed a different pattern. Two distinct behavioral phases appear to be separated by June 2020, following the onset of the COVID-19 pandemic. Prior to that date, consumption declined relative to the first segment (from 0.792 to 0.643 DID). In the subsequent segment, consumption further decreased from 0.643 to 0.480 DID, although with substantially greater monthly variability (21.1% vs. a mean of 8.5% during the first two segments). This increase in variability during the third segment may be related to post-pandemic effects on healthcare utilization and fluctuations in the number of treated patients.

It might be hypothesized that tourism—the main economic activity of the Canary Islands—could have influenced IRF consumption. In 2020, due to mobility restrictions associated with the COVID-19 pandemic, visitor numbers to the province of SCTF declined sharply, from 6.2 million tourists in 2018 to 1.8 million in 2020. By 2022, with 5.8 million visitors, pre-pandemic levels had nearly been restored (Turismo de Islas Canarias 2025). However, this marked decline in 2020 and subsequent recovery through 2022 is not reflected in the consumption curves (Figs. 2 and 3).

Furthermore, patients receiving IRF for BTcP—typically occurring in advanced or terminal stages of cancer—are unlikely to constitute a substantial proportion of the tourist population. It is also highly probable that such patients travel with their medication, given the strict prescription controls governing these products. For these reasons, tourism is unlikely to have had a meaningful impact on IRF consumption.

Self-medication without a physician’s indication was ruled out, as these medicines are subject to mandatory medical prescription (Ministry of the Presidency 2012). Iglesias-Carbajo (2017) studied various prescriber-related variables (age, sex, employment status—permanent contract or temporary/substitute, rural or urban location of the medical practice, and number of patients per physician), and did not find any factor that explained the variability in prescribing response to medicines affected by safety alerts. Therefore, we believe that prescribing habits play an important role in the degree of adherence to regulatory measures.

Our findings show an undeniable decline in annual IRF consumption between 2018 and 2022, greater in the province of SCTF (almost 55%) compared with the national decrease (34%), which shows that the intervention of the regional health administration contributed to the objective of rationalizing IRF use, even with greater effectiveness than the national intervention. Rubio-Esparza et al. (2025) also referred to the possible positive effect of measures approved by regional authorities in moderating consumption since 2017.

Torres-Bueno et al. (2024) found that between 2018 and 2020 in the province of Salamanca (Spain) there was a slow increase in oral fentanyl consumption, followed by a decline, which was more pronounced in 2021. The difference with our results lies in the fact that in SCTF the decline in consumption began earlier, probably due to the effect of the action implemented by the Canary Islands health authorities.

Regarding the route of administration, oral IRF presentations were preferred over nasal ones throughout the study period in SCTF, in Salamanca, and at the national level (AEMPS 2025; Torres-Bueno et al. 2024).

Traditionally, the oral route is the most widely used and preferred by patients. Sublingual presentations (Abstral®, Avaric®, Fenticerta® EFG, and Kaptic® EFG), lozenges with applicator (Abfentiq® and Actiq®), buccal soluble film (Breakyl®), or buccal tablets (Effentora®, Fentanilo Aristo® EFG) require little or no patient collaboration for dissolution. However, nasal fentanyl formulations (Instanyl® and Pecfent®) have specific characteristics that may hinder their use by patients, such as priming the spray before first use or after several days without use, or discarding the container (Pecfent®) if 60 days or more have passed since opening (requiring the date of first use to be recorded) (AEMPS 2025; Blázquez-Puerta et al. 2020). These characteristics may influence the preference for oral formulations among physicians, patients, and caregivers.

Finally, González-Bermejo et al. (2024) found a strong linear correlation between IRF use and cancer. As noted by AEMPS in 2018, and in view of our results, it is likely that in the province of SCTF these medicines were being used outside of the authorized indication.

The results of this study demonstrate that the implementation of regulatory measures by both regional and national health administrations has been effective in the long term in reducing consumption.

As a continuation of this work, we suggest analyzing the evolution of cases of abuse and dependence reported to the SEFV related to IRF use, to verify whether they actually decrease, as well as studying the evolution of the consumption of other opioids to determine whether there has been a shift in prescribing toward these therapeutic alternatives.

### Strengths and limitations

Most drug consumption studies use data from the public health system. Our study uses real consumption data (public and private) obtained from wholesalers supplying medicines to community pharmacies. However, the information does not include demographic data (age and sex) or clinical data of the patients, nor does it allow knowledge of the indications or adverse effects related to the dispensed prescriptions. Nevertheless, with these data it was possible to reliably analyze the geographical distribution of IRF consumption.

When comparing total consumption (public and private) in the province with national consumption (public only), there is no risk of overestimating provincial consumption, or if so, the risk is minimal, since these medicines are generally expensive and unaffordable for the vast majority of patients, who therefore resort to the public healthcare system to obtain them, thus reducing bias. Moreover, the Spanish public healthcare system covers 96.5% of the population.

The study presented here is novel, as no other studies were found analyzing the impact of regulatory measures implemented by health authorities on IRF consumption in the outpatient setting at a subnational level. Furthermore, the methodology used allows for a detailed monthly analysis of the impact of these interventions. However, we faced the limitation of not having comparable national-level data, as AEMPS only provides annual consumption data for sublingual, buccal, and nasal fentanyl formulations.

Additionally, DID is an approximate measure of consumption and does not represent actual use, since the DDD is a consensus-based value derived from the average maintenance daily dose of a drug for its main indication in adults. Moreover, it is not possible to quantify packages that are partially or entirely unused by patients after being dispensed from community pharmacies. Nevertheless, the data provided by wholesalers for this study do account for returns of undispensed packages from these establishments.

The analysis of the effect of the AEMPS safety alert on consumption is incomplete, as we were unable to compare IRF use before and after February 2018 due to the lack of community pharmacy supply data prior to that date.

The negative impact of the COVID-19 pandemic on the healthcare system and patient care in 2020 was significant and could have influenced IRF consumption to some extent. However, the linear regression analysis did not detect significant changes in the trend during that year, except as previously noted for the island of La Palma.

## Conclusions

Our findings indicate that annual IRF consumption in the province of SCTF decreased by 55% between 2018 and 2022, a percentage higher than that observed at the national level. We observed a limited immediate effect on IRF prescribing after the first alert, and when more restrictive measures were implemented, the reduction was very marked and immediate. The intervention of the regional health administration immediately contributed to the objective of reducing IRF use in the province, an effect that was not observed at the national level.

Uneven behavior was observed among the islands, which may be due to their different demographic, economic, cultural, and even geographic structures.

Prescribing habits may play an important role in the degree of adherence to regulatory measures.

It is likely that in the province of SCTF, IRF was being used outside of the indication authorized by AEMPS.

## Data Availability

The data that support the findings of this study are available from COFARTE and COFARES, but restrictions apply to the availability of these data, which were used under license for the current study, and so are not publicly available. Data are however available from the corresponding author upon reasonable request and with permission of COFARTE and COFARES.
